# Supplementary material for: Chemical zymogens for the protein cysteinome
Source: Nat Commun. 2022 Aug 18;13:4861. doi: 10.1038/s41467-022-32609-1 (PMC9388531; doi:10.1038/s41467-022-32609-1)
Supplement: Supplementary file 1 — Supplementary information [file 41467_2022_32609_MOESM1_ESM.pdf]

Supplementary Information:

Chemical zymogens for the protein cysteinome

A.N. Zelikin et al.

1. Department of Chemistry and iNano Interdisciplinary Nanoscience Centre
   Aarhus University
   Aarhus C 8000
   Denmark

   Email: zelikin@chem.au.dk

2. School of Pharmacy, Queen's University Belfast, United Kingdom

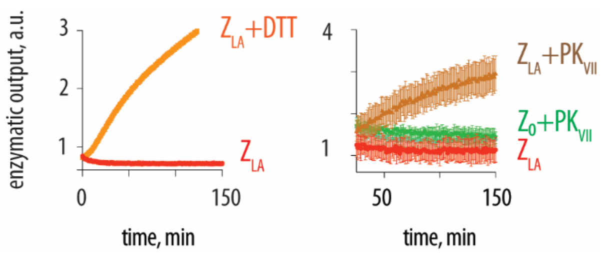

Supplementary Figure 1. Reactivation of Z_{LA} and Z_0 in PBS (pH 7.4) using DTT (left) or PK_{VII}. Reactivation of papain zymogens Z_0 and Z_{LA} was studied in physiological pH. Solutions containing papain zymogens (Z_0, = 1 µM, Z_{LA} = 0.3 µM as determined by UV absorbance at 280 nm) were combined with 10 µM of substrate and either DTT (2 mM) or PKVII (1 µM) in PBS (50 mM, pH 7.4) in a volume of 100 µL. Samples without DTT were used as controls. Fluorescence ($\lambda_{ex}/\lambda_{em}$ 370/460 nm) increase upon hydrolysis of Nα-benzoyl-L-arginine-7-amido-4-methylcoumarin was monitored at 1 min intervals at 37ºC in a plate reader. These experiments were performed in 3 independent replicates (independent zymogen syntheses).
